# Association Between Asymptomatic Vulnerable Carotid Plaques and Cognitive Impairment in Rural Adults

**DOI:** 10.3389/fneur.2020.00662

**Published:** 2020-07-07

**Authors:** Jia Zhang, Zixuan Wang, Mingyue Zhou, Jiaokun Jia, Yanfang Liu, Anxin Wang, Mengyi Guo, Shengyun Chen, Xingquan Zhao

**Affiliations:** ^1^Department of Neurology, Beijing Tiantan Hospital, Capital Medical University, Beijing, China; ^2^China National Clinical Research Center for Neurological Diseases, Beijing Tiantan Hospital, Capital Medical University, Beijing, China; ^3^Center of Stroke, Beijing Institute for Brain Disorders, Beijing, China; ^4^Department of Infectious Diseases, Beijing Tiantan Hospital, Capital Medical University, Beijing, China; ^5^Department of Neurology, Beijing Sanbo Brain Hospital, Capital Medical University, Beijing, China

**Keywords:** cognitive impairment, asymptomatic vulnerable carotid plaques, atherosclerosis, epidemiology, risk factors

## Abstract

**Background:** The prevalence of cognitive impairment is growing and higher in rural areas. The association between carotid plaque and cognitive impairment remains uncertain, and few studies focused on the cognitive function of the rural population. We designed this study to investigate the association between carotid plaque and cognitive impairment in a rural community.

**Methods:** We enrolled 3,336 participants who underwent carotid ultrasound and cognitive function measurements, free of cerebrovascular diseases, and without neurological deficits, from the China National Stroke Screen Survey program. Cognitive function was evaluated by the Montreal Cognitive Assessment (Beijing version). We used multivariable logistic regression to assess the association between asymptomatic carotid plaques and the presence of cognitive impairment.

**Results:** Nine hundred seventy-six participants had cognitive impairment in this study. After adjustment for potential confounding factors, asymptomatic carotid plaques (odds ratio was 1.35; 95% confidence interval, 1.15–1.58), especially vulnerable carotid plaques (odds ratio was 1.54; 95% confidence interval, 1.28–1.85), were associated with cognitive impairment.

**Conclusion:** In this community-based and observational study, asymptomatic vulnerable carotid plaque is an independent and significant risk factor for cognitive impairment in rural residents.

## Introduction

With society aging, the prevalence of dementia is growing. In China, around 5.6% of individuals aged ≥65 years old have dementia (1), which accounts for ~25% of the total number of dementia patients worldwide (2). This senile disease creates enormous challenges for families and society. Meanwhile, with gradually increased prevalence, cognitive impairment, which involved deteriorations in several fields of cognition, is a risk factor for dementia, disability, and other neuropsychiatric diseases (3). In China, the prevalence of dementia is higher in rural areas than in urban areas (4). However, in county hospitals, identification and establishment diagnoses of dementia are even more difficult for lack of neuropsychological experts (2). That means cognitive dysfunction of rural areas and potential risk factors need to be cared for, identified, and managed.

Carotid atherosclerosis is a well-known cause of stroke, and asymptomatic carotid atherosclerosis has a high prevalence in middle-aged and older people (5). Patients with stroke or stroke-related risk factors (such as hypertension, diabetes mellitus) could have cognitive problems (6). So, more and more researchers have focused on the impact of carotid atherosclerosis on cognition. Researchers found that carotid intima-media thickness was a risk factor for cognitive decline (7), but they had a conflict on whether carotid plaques were associated with cognitive impairment (8, 9). Besides, few studies have assessed the association between asymptomatic carotid plaque and cognitive dysfunction in rural China, and few studies have focused on the effect of plaque stability on cognitive function. Early detection of cognitive morbidity in Chinese rural community is of great significance for public health. Thus, the association between asymptomatic carotid plaque and cognitive decline, and the effect of plaque features on cognitive function need to be explored. We conducted this sizeable community-based study to investigate the impact of asymptomatic carotid plaque, especially the effect of plaque stability, on cognitive impairment in a rural population with a high risk of dementia in China.

## Methods

### Study Population

The study is a community-based and observational study in rural Chinese adults. The population was part of the China National Stroke Screen Survey (CNSSS) for the period between September 2015-September 2017. The CNSSS is an ongoing governmental program which is administered by the National Project Office of Stroke Prevention and Control (details can be found at the official website: http://cnstroke.com/WebManage/InterveneProject/Index). The survey is carried out in 31 provinces of China (not including Hong Kong, Macau, or Taiwan) among residents and aims to stroke prevention and control. Firstly, researchers selected 200 project areas in proportion to the local population size and the numbers of total counties. And then, according to geographical locations and suggestions from local hospitals, an urban community and a rural community were selected from each area as primary units. A two-stage cluster sampling method was used in every primary sampling unit. All residents aged ≥40 years of the selected communities were invited to join this study during the primary screening. The protocol of the CNSSS has been described in detail previously (10). From September 2015 to September 2017, subjects from rural communities (Beiqijia community, Changping District, Beijing and Konggang community, Shunyi District, Beijing) were enrolled in this survey. The inclusion criteria were as follows: (1) ≥40 years old, (2) target community residents as well as living in the community for at least half of a year before the survey date. A total of 5,188 residents were enrolled. Then we excluded 1,852 subjects who meet the exclusion criteria. Finally, 3,336 subjects were included in the present study. The exclusion criteria were as follows: (1) a history of stroke or TIA, (2) presence of neurologic deficits for the previous stroke, (3) a history of psychosis or schizophrenia disease, (4) other factors could interfere cognitive assessment, such as hearing loss, visual impairment, difficult to cooperate. All subjects underwent cognitive assessment, laboratory assessment, and carotid duplex sonography besides a standard questionnaire. The database was comprised of all the above information. The standard questionnaire included demographic information (such as age, sex, education, and occupation), modifiable lifestyle risk factors (such as smoking, drinking, and physical inactivity), medical history (such as hypertension, diabetes mellitus, and dyslipidemia), family history and other essential information (5). All the staff involved were trained and evaluated by the CNSSS. Senior investigators were responsible for the quality control process of screening and data entry. Researchers entered data into a database in a double-blind manner using EpiData 3.1. Written informed consent had been obtained from each participant. The study was performed according to the ethical guidelines of the 1975 Declaration of Helsinki and was approved by the Ethics Committee of Xuanwu Hospital and Beijing Tiantan Hospital.

### Assessment of Cognitive Function

Cognitive function was measured by the Montreal Cognitive Assessment (MoCA) (Beijing version). MoCA is a screening tool with high sensitivity and specificity in detecting cognitive decline (11, 12). The assessment consists of a 30-point test, including visuospatial/executive function, naming, attention, language, abstraction, memory, and orientation. MoCA score needs to be added 1 point when the subject is educated 12 years or less if the total score is <30 points (13). The previous study found that MoCA cut-off value to detect cognitive impairment was 21/22, at which the sensitivity and specificity were 68.7 and 63.9%, respectively (14). So, in the present study, cognitive impairment was defined as an overall MoCA score <22 points.

### Assessment of Carotid Ultrasound

Carotid ultrasound examinations were performed by qualified ultrasound technologists using the iU22 (Philips Healthcare) with a 6 to 10 MHz linear array transducer. All of the ultrasound technologists received unified training by ultrasound experts and passed the theoretical and practical exams before participating in the CNSSS project. A fixed ultrasound expert group was responsible for the on-site supervision of the examination and the quality control of results. After quality control, ultrasound test results could be entered into the database. All procedures were performed in accordance with the Chinses stroke vascular ultrasound examination guidelines (15). We detected plaques on each common carotid artery, bifurcation of the common carotid artery, and internal carotid artery, both near and far walls of these arterial parts were detected in longitudinal and transverse planes. All the detailed procedures were described previously (5). Carotid plaque was defined as a focal thickening of at least 50% greater than surrounding wall thickness or an intima-media thickness ≥ 1.5 mm. The stability of carotid plaque was determined via carotid ultrasound based on the morphology of plaque. Ultrasonographers recorded surface, echogenicity, and plaque texture during the examination. A vulnerable plaque was defined as anechogenic or heterogeneous plaque or plaque with ulcerative or irregular surfaces (15).

### Potential Covariates Assessment

We used standard questionnaires to collect demographic information (e.g., age, sex, education), smoking and drinking status, and medical history. We recorded weight and height, and calculated body mass index. Smoking and drinking status were classified as “never” and “current,” according to self-reported information. The participants needed to ask the frequency of drinking (“never,” “hardly ever,” or “≥ 3 times per week”). Hypertension was defined as a self-reported history, systolic blood pressure ≥140 mmHg, diastolic blood pressure ≥90 mmHg, or taking antihypertensive medications (16). Diabetes mellitus was defined as a self-reported history, fasting blood glucose level ≥7.0 mmol/L, or any use of insulin or oral hypoglycemic agents (16). Dyslipidemia was defined as the presence of a history of dyslipidemia, triglycerides ≥1.7 mmol/L, total cholesterol ≥5.17 mmol/L, low-density lipoprotein cholesterol ≥3.37 mmol/L, high-density lipoprotein cholesterol <1.04 mmol/L, or treated with lipid-lowering medications (16). Myocardial infarction and atrial fibrillation were defined as self-reported histories. Hyperhomocysteinemia was identified as plasma homocysteine level >15 μmol/L (17).

### Statistical Analysis

Statistical analysis was performed using Statistic Package for Social Science (SPSS) 23.0 for mac (IBM-SPSS, Chicago, Illinois, USA). Because the number of drinking ≥ 3 times per week group was small (*n* = 86), we treated this group together with “hardly ever.” So, the classified of the frequency of drinking was the same as the drinking status. Continuous variables were presented as mean and standard deviation (SD) (for normally distributed), or medians and interquartile ranges (IQR) (for non-normally distributed). Categorical variables were presented as frequencies and percentages. Depending on the distribution of continuous variables, we used Student's *t-*test, Mann-Whitney test, or Kruskal-Wallis test to compare them. The chi-squared test or Fisher's test was used for categorical variables. We used logistic regression to assess the association between asymptomatic carotid plaques and the presence of cognitive impairment, and calculated odds rates (ORs) and 95% confidence intervals (CIs). All the statistical tests were two-sided, and a *p*-value < 0.05 was considered to be statistically significant.

## Results

Of 3,336 participants included, 1,446 (43.3%) had carotid plaques, 874 (26.2%) were detected with vulnerable plaques, and 572 (17.1%) with stable plaques, 976 (29.3%) subjects were identified having cognitive impairment. Of all subjects, the mean age was 58.43 ± 8.43 years, 1,546 (46.3%) were male, 1,790 (53.7%) were female, and the median score of the MoCA was 24 (IQR: 21, 27).

The overall clinical characteristics of the study population, according to the MoCA score, are presented in Table 1. There were significant differences in age, education, drinking, history of hyperhomocysteinemia, presence of asymptomatic carotid plaques, and vulnerable carotid plaques between two groups. Compared with subjects with MoCA score ≥22 points (intact cognition), subjects with MoCA score <22 points (cognitive impairment) were older (60.84 ± 8.04 years vs. 57.44 ± 8.39 years, *p* < 0.001), lower educated (ratio of 12 years of education or less: 97.5 vs. 93.0%, *p* < 0.001), having higher prevalence of hyperhomocysteinemia (48.8 vs. 43.9%, *p* = 0.010), having more presence of asymptomatic carotid plaques (51.0 vs. 40.2%, *p* < 0.001), and vulnerable carotid plaques (33.3 vs. 23.3%, *p* < 0.001), and have lower prevalence of drinking (23.3 vs. 26.6%, *p* = 0.040).

**Table 1:** Clinical characteristics of study population according to the MoCA score.

**Characteristics**	**Total *n =* 3336**	**MoCA ≥ 22 *n =* 2360 (70.7%)**	**MoCA <22 n = 976 (29.3%)**	***p***
Age, years	58.43 ± 8.43	57.44 ± 8.39	60.84 ± 8.04	<0.001
Sex, n (%)				0.360
Male	1,546 (46.3)	1,106 (46.9)	440 (45.1)	
Female	1,790 (53.7)	1,254 (53.1)	536 (54.9)	
Education, n (%)				
≤ 12 years	3,147 (94.3)	2,195 (93.0)	952 (97.5)	<0.001
>12 years	189 (5.7)	165 (7.0)	24 (2.5)	
Smoking, n (%)	868 (26.0)	621 (26.3)	247 (25.3)	0.573
Drinking, n (%)	854 (25.6)	628 (26.6)	226 (23.2)	0.040
Hypertension, n (%)	1,074 (32.2)	744 (31.5)	330 (33.8)	0.207
Dyslipidemia, n (%)	2,401 (72.0)	1,718 (72.8)	683 (70.0)	0.107
Diabetes mellitus, n (%)	682 (20.5)	464 (19.7)	219 (22.4)	0.073
BMI, n (%)				0.198
<30 kg/m^2^	2,891 (86.7)	2,057 (87.2)	834 (85.5)	
≥30 kg/m^2^	445 (13.3)	303 (12.8)	142 (14.5)	
Heart diseases, n (%)	204 (6.1)	139 (5.9)	65 (6.7)	0.427
Hyperhomocysteinemia, n (%)	1,512 (45.3)	1,036 (43.9)	476 (48.8)	0.010
Carotid plaques, n (%)	1,446 (43.3)	948 (40.2)	498 (51.0)	<0.001
Vulnerable carotid plaques	874 (26.2)	549 (23.3)	325 (33.3)	<0.001

*Data are presented as mean (SD), or N (%)*.

*No., number; MoCA, Montreal Cognitive Assessment (Bejing version); BMI, body mass index*.

Table 2 shows the association between asymptomatic carotid plaques and cognitive impairment in regression models. In univariate logistic regression analysis, the asymptomatic carotid plaque was associated with the presence of cognitive impairment, OR value was 1.55 (95% CI, 1.34–1.80), *p* < 0.001. After adjusting for age, sex, education level, and other potential confounding factors, the carotid plaque was still an indicator of cognitive impairment, multivariate-adjusted OR value was 1.35 (95% CI, 1.15–1.58), *p* < 0.001. Then we divided all subjects into three groups according to plaque stability and performed analyses. Results indicated that vulnerable plaque was associated with cognitive impairment after adjusting for any possible confounding factors, the multivariate-adjusted OR value was 1.54 (95% CI, 1.28–1.85), *p* < 0.001.

**Table 2:** Odds ratios for cognitive impairment according to asymptomatic carotid plaques.

	**Carotid plaques**				
	**No OR (95% CI)**		**Yes OR (95% CI)**		***p***
Crude model	Reference		1.55 (1.34–1.80)		<0.001
Model 1	Reference		1.32 (1.12–1.54)		0.001
Model 2	Reference		1.34 (1.14–1.57)		<0001
Model 3	Reference		1.35 (1.15–1.58)		<0.001
	**Stability of carotid plaques**				
	**No OR (95% CI)**	**Stable plaques OR (95% CI)**	***p***	**Vulnerable plaques OR (95% CI)**	***p***
Crude model	Reference	1.28 (1.04–1.57)	0.019	1.75 (1.47–2.08)	<0.001
Model 1	Reference	1.10 (0.89–1.37)	0.373	1.53 (1.28–1.84)	<0.001
Model 2	Reference	1.09 (0.88–1.35)	0.443	1.53 (1.28–1.84)	<0.001
Model 3	Reference	1.09 (0.88–1.36)	0.416	1.54 (1.28–1.85)	<0.001

*OR, odds ratio; CI, confidence interval*.

*Model 1, adjusted for age, sex, and education; Model 2, adjusted for age, sex, education, drinking, diabetes mellitus, and hyperhomocysteinemia; Model 3, adjusted for age, sex, education, smoking, drinking, hypertension, dyslipidemia, diabetes mellitus, heart diseases, hyperhomocysteinemia, and body mass index*.

Further analyses of the interaction effects in predefined subgroups were shown in Table 3. There was no evidence of significant interaction effects on the association between asymptomatic vulnerable carotid plaques and cognitive impairments in any predefined subgroups (all p for interaction > 0.05) though heart disease showed a trend for significance.

**Table 3:** Association between cognitive impairment and asymptomatic carotid plaques in predefined subgroups.

**Subgroup**	**Stability of carotid plaques**					
	**No plaques OR (95% CI)**	**Stable plaques OR (95% CI)**	***p***	**Vulnerable plaques OR (95% CI)**	***p***	***P* interaction**
Age						0.203
<60 years	Reference	1.37 (0.99–1.90)	0.055	1.58 (1.19–2.11)	0.002	
≥60 years	Reference	0.93 (0.69–1.24)	0.621	1.52 (1.17–1.96)	0.001	
Sex	Reference					0.819
Male	Reference	1.19 (0.87–1.63)	0.265	1.66 (1.26–2.17)	<0.001	
Female	Reference	1.03 (0.76–1.40)	0.847	1.52 (1.17–1.99)	0.002	
Education	Reference					0.831
≤ 12 years	Reference	1.11 (0.89–1.38)	0.359	1.58 (1.30–1.91)	<0.001	
>12 years	Reference	0.93 (0.20–4.24)	0.929	1.46 (0.48–4.23)	0.509	
Smoking	Reference					0.134
Yes	Reference	1.32 (0.87–1.98)	0.188	1.28 (0.88–1.84)	0.192	
No	Reference	1.02 (0.79–1.33)	0.861	1.74 (1.39–2.16)	<0.001	
Drinking	Reference					0.741
Yes	Reference	1.20 (0.77–1.86)	0.426	1.69 (1.16–2.46)	0.007	
No	Reference	1.09 (0.85–1.40)	0.497	1.53 (1.23–1.91)	<0.001	
Hypertension	Reference					0.433
Yes	Reference	1.26 (0.87–1.82)	0.224	1.81 (1.31–2.50)	<0.001	
No	Reference	1.04 (0.79–1.37)	0.771	1.49 (1.18–1.89)	0.001	
Dyslipidemia	Reference					0.896
Yes	Reference	1.13 (0.87–1.45)	0.359	1.55 (1.24–1.93)	<0.001	
No	Reference	1.04 (0.68–1.59)	0.858	1.74 (1.20–2.52)	0.003	
Diabetes mellitus	Reference					0.828
Yes	Reference	0.94 (0.59–1.51)	0.802	1.54 (1.04–2.27)	0.029	
No	Reference	1.15 (0.90–1.47)	0.261	1.59 (1.28–1.97)	<0.001	
Heart diseases	Reference					0.062
Yes	Reference	2.88 (1.06–7.81)	0.038	2.75 (1.18–6.43)	0.019	
No	Reference	1.04 (0.83–1.30)	0.753	1.53 (1.26–1.8)	<0.001	
HHCY	Reference					0.206
Yes	Reference	0.96 (0.70–1.30)	0.780	1.65 (1.26–2.15)	<0.001	
No	Reference	1.30 (0.96–1.76)	0.095	1.48 (1.13–1.94)	0.004	
BMI	Reference					0.914
<30 kg/m^2^	Reference	1.11 (0.88–1.41)	0.367	1.57 (1.28–1.93)	<0.001	
≥30 kg/m^2^	Reference	1.08 (0.60–1.97)	0.790	1.68 (1.01–2.80)	0.045	

*OR, odds ratio; CI, confidence interval; Ref, reference; HHCY, hyperhomocysteinemia; BMI, body mass index*.

*Adjusted for age, sex, education, smoking, drinking, hypertension, dyslipidemia, diabetes mellitus, heart diseases, hyperhomocysteinemia, and body mass index*.

## Discussion

In this large community-based study, we investigated the cognitive function of rural adults and found that the presence of cognitive impairment was 29.3%. The main results of our study were that asymptomatic carotid plaques, especially the vulnerable plaques, were associated with a decline in cognition. We did not find that age, sex, education, smoking, drinking, hypertension, dyslipidemia, diabetes mellitus, heart disease (myocardial infarction, and atrial fibrillation), hyperhomocysteinemia, or BMI had effects on the association between vulnerable plaques and cognitive decline.

In Chinese rural areas, in addition to having a higher prevalence of cognitive impairment, the prevalence of atherosclerotic carotid disease is also higher (41.6%) than in urban areas (30.8%) (5). In our study, we focused on both cognitive impairment and carotid atherosclerosis and explored the relationship between asymptomatic carotid plaque and cognitive dysfunction. To our knowledge, our study is the first study to provide evidence for the association between asymptomatic vulnerable carotid plaques and cognitive function in Chinese rural people. Based on our results, medical professionals need to pay more attention to rural patients with asymptomatic vulnerable carotid plaques to early identify cognitive impairment and prevent the development of the decline in cognition.

Several researches have associated carotid atherosclerosis with cognitive impairment, but these researches only evaluated the presence and some plaque features including site, number, and total plaque area of carotid plaque (8, 18–20). However, some studies controversially found that carotid plaque was not a risk factor for cognitive impairment (21, 22). Note that none of the above studies evaluated the stability of carotid plaques. We found the conflict results of these studies may be caused by that they did not assess the stability of carotid plaques. A few studies (23–25) focused on the stability of carotid plaques and indicated the association between vulnerable plaques and cognitive impairment. Our results were in line with them. Our study explored the effects of both the presence and stability of carotid plaque on cognitive function and favored the association between vulnerable carotid plaque and the decline in cognition.

There are some potential mechanisms that may explain the relationship between carotid plaque and cognitive impairment. Rupture of vulnerable carotid plaque followed by thromboembolism is one of the major causes of ischemic stroke (26), and the cerebral emboli are believed to be one of the potential causes of cognitive impairment (27). Besides, carotid atherosclerosis could decrease cerebral perfusion pressure. Cerebral hypoperfusion may contribute to brain dysfunction, which was related to the onset of clinical dementia (28). All of these may impair brain structure. Studies found that silent brain infarction may play an intermediate role in atherosclerotic carotid disease and cognitive impairment (29). The silent brain infarcts increased the risk of dementia (30). Carotid plaques were also associated with white matter lesions, which were also the pathogenesis of cognitive impairment (31, 32). Furthermore, carotid atherosclerosis, especially vulnerable plaque, may be a marker of inflammation and endothelial dysfunction. These pathogenic pathways may cause brain atrophy, which could lead to cognitive impairment (33, 34).

Different from previous studies assessing several specific cognitive domains or evaluating general cognition by mini-mental state examination (MMSE), we used MoCA to measure general cognitive function. MoCA covers more cognition domains and is more sensitive in identifying mild cognitive impairment compared with MMSE (12). Using MoCA is good for detecting cognitive decline earlier. In our study, we identified a higher prevalence of cognitive decline in Chinese rural areas via MoCA, and people with vulnerable carotid plaques were at high risk of cognitive impairment. Community doctors and local health systems need to pay more attention to the cognitive function of rural populations with asymptomatic vulnerable carotid plaque. Early identification and intervention of asymptomatic vulnerable carotid plaque are necessary to prevent the progression of cognitive decline.

The strength of the present study includes large sample size and focusing on the stability of carotid plaques. Several limitations of our study need to be noted. We used the ultrasound examination and did not assess the thickness of the plaques. Further studies need to assess the thickness of the plaques and use more accurate examination, such as high-resolution magnetic resonance imaging (MRI), to validate our findings. We did not do the cranial MRI test, which could detect such as silent embolic infarcts, white matter lesions, brain atrophy, and evaluate the vertebrobasilar system. Besides, education years affect cognitive assessment performance. We need to collect more detailed information on education to confirm our results in future studies. The present study showed a potential trend of an interaction of heart disease with vulnerable carotid plaques on cognitive impairment, which was needed to be confirmed in future studies. Our study was conducted in Asians. More studies, including other races, ethnicities, and nations, are needed to confirm our findings. All of these limitations need to be addressed in future studies.

## Conclusion

In conclusion, the study results suggest that asymptomatic vulnerable carotid plaque is an independent and significant risk factor for cognitive impairment in rural Chinese population.

## Data Availability Statement

The datasets generated for this study are available on request to the corresponding author.

## Ethics Statement

The studies involving human participants were reviewed and approved by Xuanwu Hospital and Beijing Tiantan Hospital. The patients/participants provided their written informed consent to participate in this study.

## Author Contributions

JZ interpreted the data and drafted the manuscript. ZW made review and editing. MZ and JJ acquired the data. YL, AW, and MG analyzed the data and prepared the manuscript. SC designed the research. XZ designed the research and handled funding and supervision. All authors contributed to the article and approved the submitted version.

## Conflict of Interest

The authors declare that the research was conducted in the absence of any commercial or financial relationships that could be construed as a potential conflict of interest.
